# Phylogenetic and Pathotypical Analysis of Two Virulent Newcastle Disease Viruses Isolated from Domestic Ducks in China

**DOI:** 10.1371/journal.pone.0025000

**Published:** 2011-09-19

**Authors:** Shouping Zhang, Xiaoting Wang, Changguang Zhao, Dehua Liu, Yanxin Hu, Jixun Zhao, Guozhong Zhang

**Affiliations:** Key Laboratory of Animal Epidemiology and Zoonosis, College of Veterinary Medicine, Ministry of Agriculture, China Agricultural University, Beijing, People's Republic of China; Virginia Polytechnic Institute and State University, United States of America

## Abstract

Two velogenic Newcastle disease viruses (NDV) obtained from outbreaks in domestic ducks in China were characterized in this study. Phylogenetic analysis revealed that both strains clustered with the class II viruses, with one phylogenetically close to the genotype VII NDVs and the other closer to genotype IX. The deduced amino acid sequence of the cleavage site of the fusion (F) protein confirmed that both isolates contained the virulent motif ^112^RRQK/RRF^117^ at the cleavage site. The two NDVs had severe pathogenicity in fully susceptible chickens, resulting in 100% mortality. One of the isolates also demonstrated some pathogenicity in domestic ducks. The present study suggests that more than one genotype of NDV circulates in domestic ducks in China and viral transmission may occur among chickens and domestic ducks.

## Introduction

Newcastle disease virus (NDV) belongs to genus *Avulavirus* in the family *Paramyxoviridae* and has also been designated as avian paramyxovirus 1 [1]. Its genome is a non-segmented, single-stranded, negative-sense RNA molecule of approximately 15,186 nucleotides (nt) that contains six genes encoding the six structural proteins (from the 3′ to 5′ terminus): nucleoprotein (NP), phosphoprotein (P), matrix (M), fusion (F), hemagglutinin-neuraminidase (HN) and the large protein (L) [2]. Additionally, two nonstructural proteins (V and W) may be generated due to an mRNA-editing event in which one (V) or two (W) G residues are inserted at a specific position within the P gene mRNA [3], [4].

NDV strains are classified as high virulence (velogenic), intermediate (mesogenic) or low virulence (lentogenic) based on some biological parameters, such as the mean death time (MDT) of chicken embryos infected with the minimum lethal dose of virus, the intracerebral pathogenicity index (ICPI) in 1-day-old chicks and the intravenous pathogenicity index (IVPI) in 6-week-old chickens. The velogenic strains are involved in fatal infections of chickens. The mesogenic strains cause moderate respiratory signs with occasional nervous signs while the lentogenic strains typically cause subclinical infections or mild respiratory disease [5], [6], [7]. The molecular basis for NDV pathogenicity is dependent on the cleavability of precursor F (F0) to active F1 and F2 polypeptides by cellular proteases [2], [8].

Phylogenetic analysis revealed that NDV strains consist of two distinct classes (class I and class II) within a single serotype. Class I viruses comprise at least nine (1–9) genotypes and have been recovered primarily from wild waterfowl and live bird markets. Class II viruses comprise the vast majority of the sequenced NDVs and include isolates recovered from poultry, pet birds and wild birds, and are further categorized into genotypes I–XI [9], [10], [11], [12], [13], [14], [15].

NDV has a wide host range with most orders of birds reported to have been infected by the virus, the more commonly affected species include chickens, turkeys, pigeons and ducks. Other species can be infected, and this occasionally includes mammals [5], [16]. Chicken infection with virulent NDVs can be devastating due to the resulting high mortality or significant egg drop, and is characterized by very rapid spread. The disease remains one of the major problems affecting existing or developing poultry industries in many countries. In general, ducks are considered natural reservoirs of NDV and show few or no clinical signs after infection even for NDV strains lethal to chickens [13], [17], [18], [19]. Many NDVs have been isolated from domestic ducks in recent years [12], [13], [20]. Most of these are low-virulence strains, occasionally a high-virulence strain is isolated but little is known about their potential to cause disease in domestic ducks.

In the present study, two velogenic NDVs obtained from outbreaks in domestic ducks in China were pathotypically and genotypically characterized. We also discuss the evolutionary relationship of NDVs from different origins.

## Results

### Biological characteristic assessment of the two isolates

As determined by the MDT and IVPI, both NDV isolates were velogenic strains. The MDT/IVPI values and other details are shown in Table 1.

**Table 1:** Details of the two NDV isolates investigated in this study.

Isolate name	Species	Province	Country	MDT^a^	ICPI^b^	EID_50_/0.1 ml
SD09	Duck	Shandong	China	55	1.675	10^4.32^
GD09-2	Duck	Guangdong	China	38	1.725	10^7.25^

a Mean death time in embryonating eggs (hours) (<60: velogen; 60–90: mesogen; >90: lentogen).

b Intracerebral pathogenicity index in day-old chicks (1.5–2.0: velogen; 1.0–1.5: mesogen; <0.5: lentogen).

### RT-PCR and sequence analysis

The RT-PCRs performed with all primers (Table 2) resulted in amplification of the expected products. The amplified products were sequenced, annotated and assembled to obtain the entire nucleotide sequences of two isolates. The nucleotide sequence data were deposited into the Genbank database and the accession numbers were HQ317394 (GD09-2) and HQ317395 (SD09). The coding regions of both strains were 14,879 nt in length. Compared with NDV Lasota, the two isolates bear a 6 nt insertion (CCCCCC or TCCCAC) in positions 1647–1648 nt of the NP gene.

**Table 2:** Primers used in the study.

Primers	Sequences(5′-3′)	Position	Expected size (bp)
1-F	TACGATAAAAGGCGAAGGAG	23–42	1122
1-R	CAGGACTGATGCCATACCC	1126–1144	
2-F	CGGAGAAGCAATCGAGATCGTAC	77–99	1501
2-R	CACTGGGTAGAAGGGAGAACAGA	1555–1577	
3-F	ACCAAGACTTCAGCCCTCG	950–968	1173
3-R	GACGGTTGTTTGTCTGGTCTGT	2101–2122	
4-F	GGAGACTTGGAGTAGAGTATGCT	1200–1222	1464
4-R	CATAGGAATGGAGGATGTCTG	2143–2663	
5-F	CTTCTACCCAGCAGACCAG	1867–1885	1157
5-R	ATCCAGCTTACTCAGGAGTTTA	3002–3023	
6-F	CAAGCAACTCCCTTCTGTCC	2230–2249	1173
6-R	TGTTTCTTCCCGTCTCCTG	3384–3402	
7-F	TAAACCTGCCACGGTAAGC	2906–2924	1785
7-R	GTCTCCCGTTACTACAATCC	4671–4690	
8-F	CCACGCTTCAACACCCAAAAC	3157–3177	1478
8-F	TCGGACGGATACAGCCCAAT	4615–4634	
9-F	TTACTTGCTCCTTTCTTCTC	4160–4179	2188
9-R	TACTCTGACCGTTCTACCC	6329–6347	
10-F	AGTCTGGGTTTAGCGTGTTA	6101–6120	2111
10-R	AGCATTATGGGAGATGATTGG	8191–8211	
11-F	GGGAAGACGACACCGCACCAATC	8174–8196	1868
11-R	CGCCCATTCACTTTCACCTCTTT	10019–10041	
12-F	TGGAATACCTGACAACCCTC	9951–9970	1882
12-R	TCTCCCTCCACAAGTTCTATG	11812–11832	
13-F	GGCAGGAAGATACTGGGTGT	11177–11196	2319
13-R	CGCAGGTTGTCGGGTAAATG	13476–13495	
14-F	CTGTGGGTAGGAGAAAGC	11523–11540	1802
14-R	CGTGATTATGTTGGGAGAC	13306–13324	
15-F	GCTGTGAGACCATTACTTAG	13262–13281	1908
15-R	ACAGAACTACACTCAAGAGC	15150–15169	
16-F	ACCTGAATGAGAAGATGCT	13116–13134	1623
16-R	TGAGACCCAGTATTGTGAC	14720–14738	
17-F	TGTGCGGAAAGTTTGGTGAC	14510–14529	539
17-R	GAGGGAGTCATCAGTTAGGAAG	15027–15048	

Proteolytic cleavage site motifs (residues 112–117) for the F0 protein in the two isolates were analyzed. Strain SD09 was shown to have a virulent motif (^112^RRQKRF^117^) composed of multibasic amino acids at the F0 cleavage site. This motif is commonly found in strains that are highly virulent in chickens, especially in genotype VII viruses [2], [21]. Strain GD09-2 exhibited the sequence motif ^112^RRQRRF^117^, which is another common motif in other virulent NDVs including strain F48E9 (a genotype IX virus that was isolated only in China).

### Phylogenetic analysis

The predicted amino acid sequences of the two isolate were compared. Nucleotide and amino acid sequence data of 63 NDV reference strains obtained from the GenBank database (Table 3) were used for comparison. The GD09-2 isolate showed greatest nucleotide and amino acid identities (99.75%) with the velogenic strain F48 (Accession number FJ436302). Strain SD09 was highly similar to GM (97.78%; Accession number DQ486859), a classic genotype VII virus. The two isolates (GD09-2 and SD09) had sequence homologies of 87.70% and 82.64% respectively at the nucleotide level with strain LaSota, the common vaccine strain used in China.

**Table 3:** NDV strains and their accession numbers used for phylogenetic analysis.

Virus strains	Year	Country	Genotype	Accession number
GM	2007	China	VII	DQ486859
Muscovy duck/China(Fujian)/FP1/02	2009	China	VII	FJ872531
Chicken/China/Guangxi9/2003	2008	China	VII	DQ485230
Chicken/China/Guangxi11/2003	2008	China	VII	DQ485231
SF02	2005	China	VII	AF473851
JSD0812	2009	China	VII	GQ849007
NA-1	2006	China	VII	DQ659677
ZJ1	2007	China	VII	AF431744
Mallard/China/HLJ-07-05	2007	China	VII	EF592500
Mallard/China/HLJ-50-06	2007	China	VII	EF592505
DFQS/Beijing/08	2010	China	VII	FJ608350
WF00D	2009	China	VII	FJ754272
WN/Tianjin/03	2010	China	VII	FJ608334/FJ608352
Goose/China/HLJ-48-06	2007	China	VII	EF592504
YZCQ/Liaoning/08	2010	China	VII	FJ608351
GM/Shandong/01	2010	China	VII	FJ608361
XD/Shandong/08	2010	China	VII	FJ608365
JAU04	2006	China	VII	EF141104
TW-03-332	2010	Taiwan,China	VII	EU526308
TW-03-333	2010	Taiwan,China	VII	EU526309
QG/Hebei/07	2010	China	VII	FJ608355
HG/Beijing/2009	2010	China	VII	FJ882015
PX2/03	2007	China	VII	EF175145
HZ	2005	China	VII	DQ114478
Taiwan/95	1996	Taiwan,China	VII	U62620
JS/1/03/Go	2008	China	VII	DQ682437
Dove/Italy/2736/00	2004	Italy	VI	AY562989
P4	2010	China	VI	HM063425
Pigeon/Italy/1166/00	2004	USA	VI	AY288996
Turkey/USA(ND)/43084/92	2004	USA	IV	AY289001
Herts/33	2005	Netherlands	IV	AY741404
Italien	2008	Italy	IV	EU293914
F48E9	2005	China	IX	AY508514/AY997298
CK/CH/GD/1/05	2008	China	VII	FJ480789
JS/1/02/Du	2009	China	IX	FJ436306
AUS32	2003	Austria	III	AF542891
D26/76	1999	Austria	I	M24692
V4	2003	Austria	I	AF542946
FJ0801	2009	China	I	FJ600541
ND-XX08	2009	China	VII	GQ853450
JS/1/04/Go	2008	China	VII	DQ682448
SD/1/04/Go	2008	China	VII	DQ682450
Ulster 2C	1994	U.K	I	Z30084
TexasG.B	1988	USA	II	M23407
B1	2000	USA	II	AF309418
MET95	2003	Japan	II	AY143159
Clone 30	2005	Germany	II	Y18898
HN0801	2009	China	II	FJ600543
Lasota	1999	Netherlands	II	AF077761
GPMV/QY97-1	1999	China	VI	AF192406
ZJ1	2007	China	VII	AF431744
JS/1/97/Ch	2009	China	IX	FJ436305
HB92	2003	China	II	AY225110
D26	1993	Japan	I	M19432
JS-1-05	2006	China	VII	DQ469830
SRZ03	2005	China	VII	DQ234584
Duck/1/05	2008	China	VII	EU649675
ZJ/1/86/Ch	2009	China	IX	FJ436303
Duck/China/SD27/2008	2010	China	Class I	FJ492893
Duck/China/SD23/2008	2010	China	Class I	FJ492892
Duck/China/SD08/2008	2010	China	Class I	FJ492891
Duck/China/SD26/2008	2010	China	Class I	FJ492894
Duck/China/08-004/2008	2008	China	Class I	EU589149

**Figure 1:**
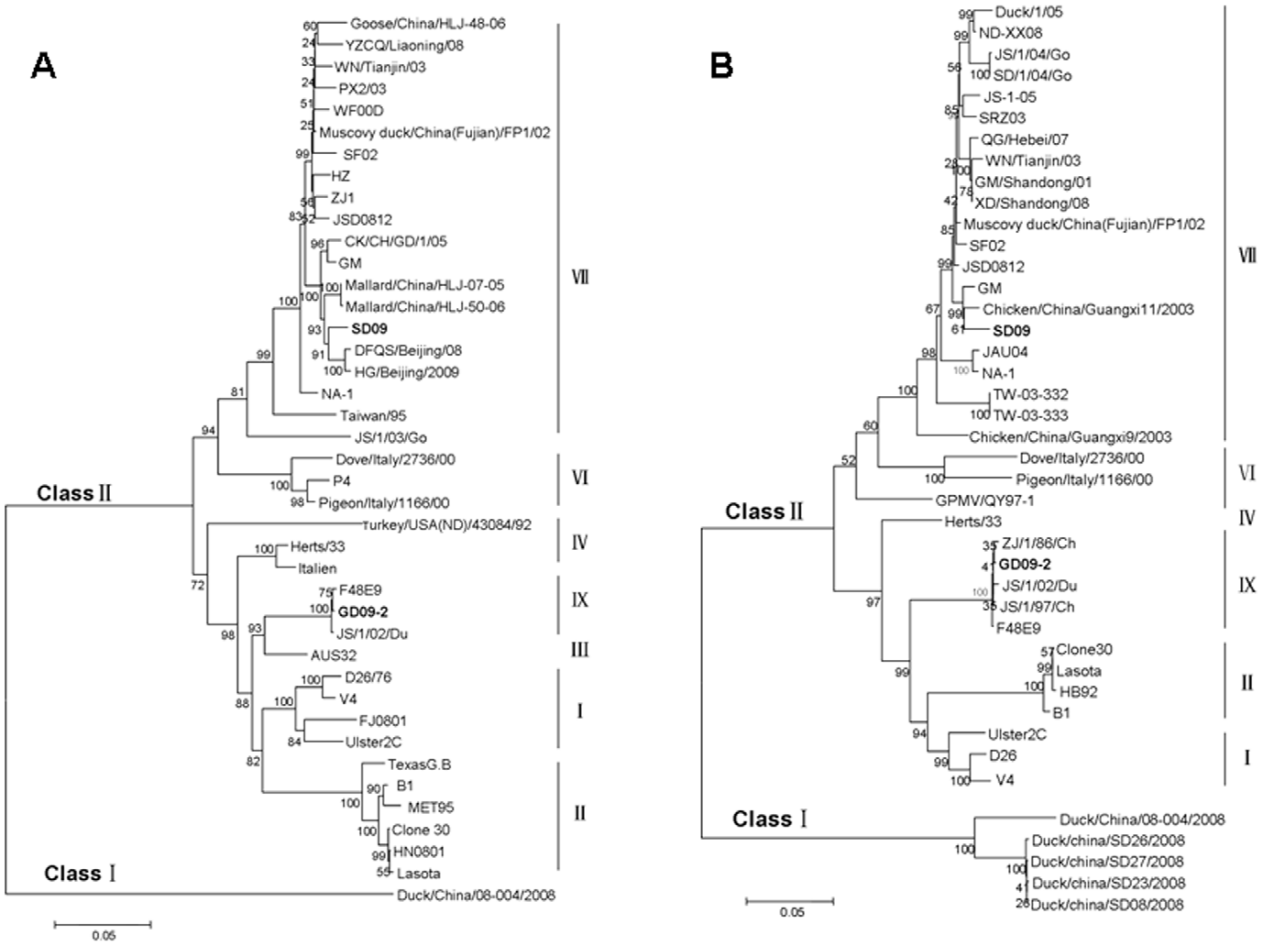
Phylogenetic tree based on the nucleotide sequences of the fusion gene (A) (nt 1–1662) and hemagglutinin-neuraminidase gene (B) (nt 1–1713 or 1731) of NDV. The phylogenetic tree was constructed by the Neighbor-Joining method with 1000 bootstrap replicates (bootstrap values are shown on the tree).

Phylogenetic analysis was conducted based on nucleotide sequences of the two important surface genes of NDV, the F and HN genes. Both isolates clustered within class II NDVs, as shown in Fig. 1. Within class II, strain SD09 was phylogenetically close to genotype VII NDVs, which displayed a high nucleotide sequence homology of 95.59–97.78%. Strain GD09-2 was phylogenetically close to genotype IX NDVs. However, both viruses were genetically distinct and phylogenetically distant from the vaccine strains (Lasota, AF077761; B1, AF309418), and clustered in different groups.

### Pathogenicity in chickens

**Figure 2:**
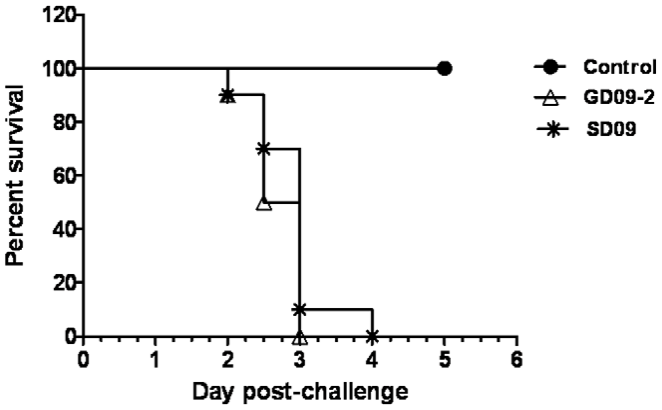
Percentage survival of chickens after inoculation with SD09 or GD09-2 velogenic NDVs.

Birds infected with SD09 or GD09-2 exhibited severe clinical disease. Slight depression and head tremor were evident in some birds at 2 dpi. Whereas at 3 dpi all birds were depressed, and some had severe nervous signs such as incoordination accompanied by leg paralysis. All infected birds were dead by 5 dpi (Fig. 2). At necropsy, severe hemorrhage could be seen in the gastrointestinal tract, especially in the proventriculus, duodenum and appendix. Hemorrhaging of liver, spleen, trachea and kidney could also be seen occasionally. Small intestine, proventriculus, spleen and kidney tissues were collected for histological observation.

**Figure 3:**
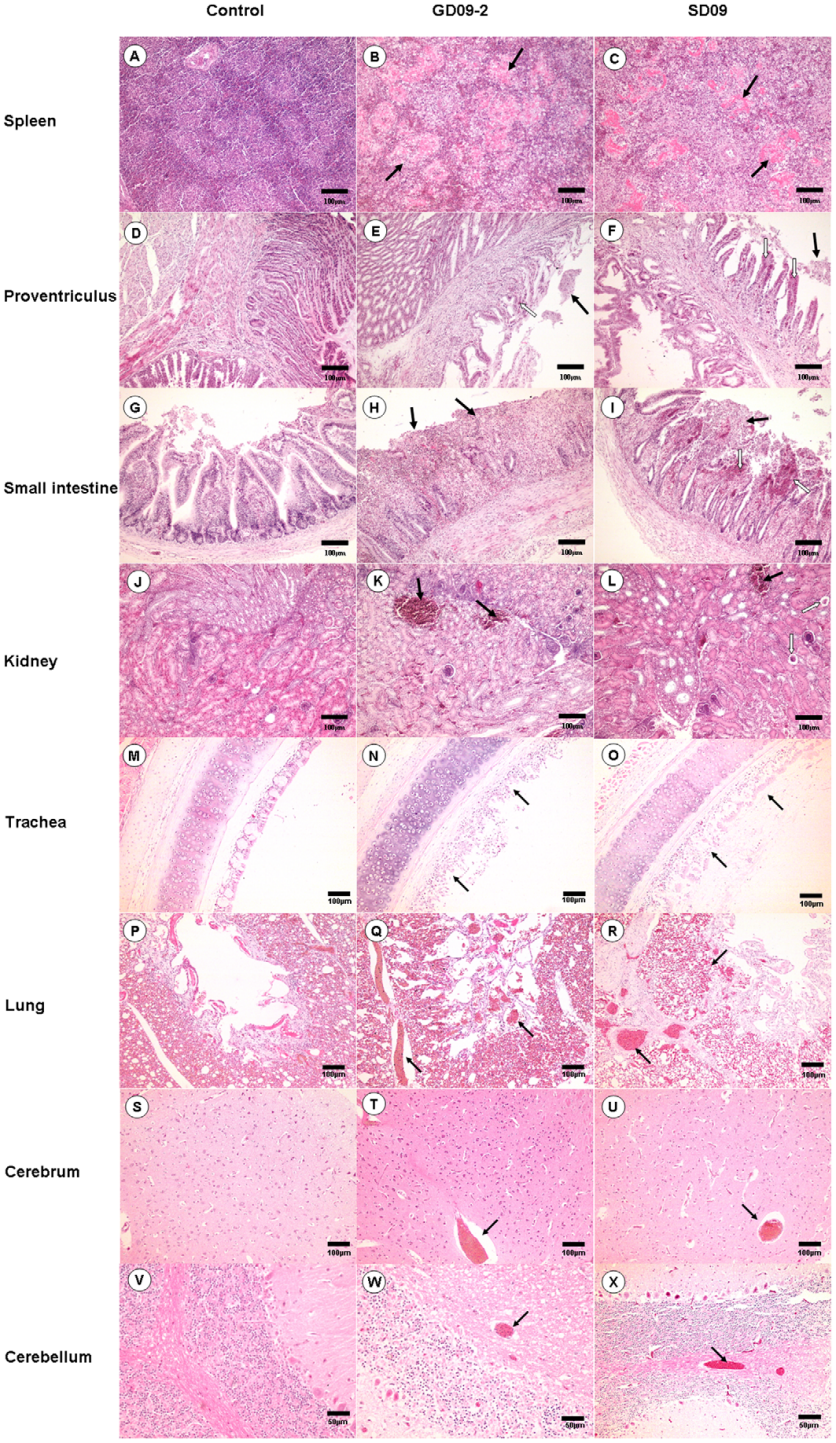
Histopathology on tissues from 1-week-old chickens infected with NDV GD09-2 or SD09 (H&E). B and C: amalgamation of collapsed cell and inflammatory exudates created the homogeneous and pink-staining appearance in the white pulps of spleens (black arrow); E and F: dropout and necrosis of the mucosal epithelia in the proventriculus (black arrow); H and I: dropout of epithelium and numerous inflammatory cell infiltration in the small intestine (black arrow); K and L: congestion (black arrow) or glomerulus atrophy (white arrow) in the kidneys; N and O: dropout and necrosis of mucous epithelial cells in the trachea (black arrow); Q and R: congestion and hemorrhage in the lung (black arrow); T and U: venous congestion in the cerebrum (black arrow); W and X: venous congestion in the cerebellum (black arrow); A, D, G, J, M, P, S and V: Corresponding control tissues. Scale bar = 50 µm in cerebellum or 100 µm in other tissues.

Chickens in the two inoculation groups exhibited histopathological changes, with the GD09-2 group displaying more severe changes than in the SD09 group. The spleen lesions exhibited a “starry sky” change, which was the result of a large number of lymphocytes disrupting and disappearing. The amalgamation of collapsed cell and inflammatory exudates created the homogeneous and pink-staining appearance of white pulps in the spleen (Fig. 3B and C). Pathological changes in the glandular stomach were hemorrhage and atrophy of the proventriculus papillae, with mucus on the surface of papillae. Dropout and necrosis of the mucosal epithelia of the proventriculus was also observed (Fig. 3E and F). The small intestine lesions of chickens infected with GD09-2 or SD-09 virus showed signs of enteritis, characterized by broken villi, dropout of epithelium and numerous inflammatory cell infiltrates (Fig. 3H and I). The lesions in the kidney were relatively slight, with congestion or glomerulus atrophy occasionally seen (Fig. 3K and L). Dropout and necrosis of mucous epithelial cells were seen in the trachea (Fig. 3N and O). In lung, congestion and hemorrhage (erythrocytes infiltrating in the pulmonary alveoli) were observed (Fig. 3Q and R). Venous congestion was present in cerebrum (Fig. 3T and U) and cerebellum (Fig. 3W and X). In comparison, all tissues from the control group had no apparent histological changes (Fig. 3A, D, G, J, M, P, S and V).

### Pathogenicity in ducks

**Figure 4:**
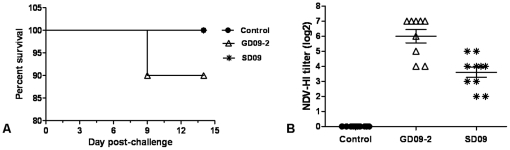
Percentage survival (A) and sero-conversion at 14 dpi (B) of ducks after inoculation with SD09 or GD09-2 velogenic NDVs.

**Figure 5:**
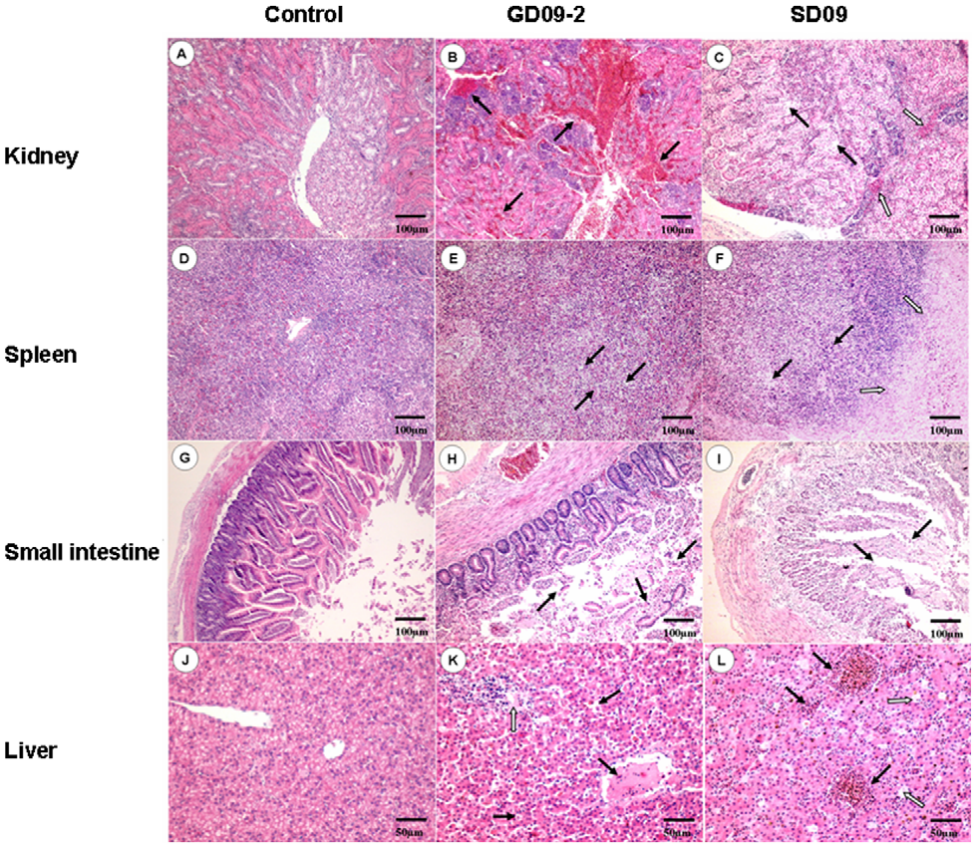
Histopathology on tissues from 1-week-old ducks infected with NDV GD09-2 or SD09 (H&E). B and C: hemorrhage (group GD09-2, black arrow) or degeneration of renal tubules epithelial cells (group SD09, black arrow) and eosinophil infiltration (group SD09, white arrow) in the kidneys; E and F: necrosis and disappear of lymphocyte (black arrow) or coagulation necrosis of massive tissue (white arrow) in the spleens; H and I: villus missing (white arrow) and necrosis of epithelial cell infiltration (black arrow) in the small intestines; K and L: dilatation of hepatic sinus and thrombus (group GD09-2, black arrow), lymphocyte infiltration (group GD09-2, white arrow), dilatation of hepatic sinus (group SD09, black arrow) and venous congestion (group SD09, white arrow) in the livers. A, D, G and J: Corresponding control tissues. A–I: scale bar = 100 µm, J–L: scale bar = 50 µm.

**Figure 6:**
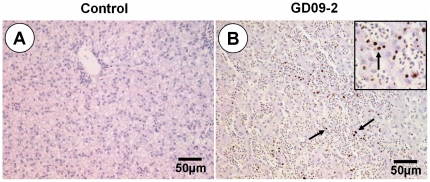
Immunohistochemical detection of NDV antigens in liver after experimental infection with NDV GD09-2. B: Viral antigen was detected extensively in the liver cells (black arrow). A: Corresponding control tissues. Scale bar = 50 µm.

No obvious clinical signs were seen in ducks within the two week observation period in the SD09 inoculation group. However, four ducks exhibited obvious clinical signs in the GD09-2 group, including depression, tears and anorexia. One duck died at 9 dpi (Fig. 4A), and bile reflux and hemorrhaging in the liver were seen at necropsy. Obvious histopathological changes could be seen in two inoculation groups including hemorrhage (Fig. 5B) and degeneration of renal tubules epithelial cells (Fig. 5C) in the kidneys, necrosis of lymphocyte and coagulation necrosis of massive tissue (Fig. 5E, F) in the spleens, villus missing and necrosis of epithelial cell infiltration (Fig. 5H, I) in the small intestines, lymphocyte infiltration, dilatation of hepatic sinus and venous congestion (Fig. 5K, L) in the livers. Using IHC, antigens of NDV were detected extensively in the liver cells in the group infected with the GD09-2 strain (Fig. 6B). Detected by RT-PCR, over 50% of the cloacal samples were positive for viral RNA on day 3, 5 and 7 dpi (Table 4). No viral RNA was detected in any of the cloacal swabs of the control ducks.

**Table 4:** Virus shedding from ducks by the cloacal route following inoculation with GD09-2 virus.

Group	No. viral RNA^a^/No. ducks tested			
	Days postinoculation			
	Day 1	Day 3	Day 5	Day 7
GD09-2	0/10	5/10	6/10	8/10
Control	0/10	0/10	0/10	0/10

a By reverse transcriptase-polymerase chain reaction.

Sera were collected at 14 dpi from surviving ducks and hemagglutination-inhibition (HI) tests were performed to detect specific NDV antibody (Fig. 4B). All sera were positive for NDV HI antibody after SD09 or GD09-2 inoculation, and the average HI titers were 6log2 and 3.6log2 respectively.

## Discussion

Outbreaks of Newcastle disease (ND) were first reported in poultry in 1926 [5]. Since then, vaccination has been widely used for prevention and control of the disease in many countries including China, but the disease is still enzootic in some areas and is recognized as major disease of poultry [2]. The prevailing NDV strains have significant differences from the current vaccine strains in their biology, serology and genetics, which might be considered as reasons for the outbreaks [2], [22], [28], [30]. In the past decade, the predominant NDV strain was genotype VII viruses in China, while the most commonly used live vaccine LaSota and Clone-30 belong to genotype II. Up to 8-fold titer differences might be observed between the vaccine and field strains on mean HI titers, which may reflect apparent antigenic differences among them.

Phylogenetic analysis reveals that NDVs are continually evolving due to immune pressure and the broad genetic diversity of NDV. Some reports have revealed that waterfowl and wild birds may play an important role in the evolution of NDV [6], [12], [23], [24], [25], [26], [27]. However, virological and epidemiological information about NDV strains circulating in waterfowl and wild birds is still extremely limited. In ducks, many NDVs have been isolated in recent years [12], [13], [17], [18], [19], [20]. Most of them belong to class I NDVs and are low-virulence strains, occasionally a high-virulence strain is isolated but little is known about their potential to cause disease in domestic ducks.

Two representative NDV isolates obtained from outbreaks in domestic ducks in China were characterized both pathotypically and genotypically in the study. Pathogenicity tests showed that both isolates (SD09 and GD09-2) were velogenic strains. They had severe pathogenicity in fully susceptible chickens, resulting in 100% mortality. The GD09-2 strain also demonstrated some pathogenicity in domestic ducks. The results revealed that ducks may not only be a natural reservoir of NDV but also become susceptible to flocks, similar to the changes that occurred with the highly pathogenic avian influenza (HPAI) over recent decades. Therefore, more attention must be paid to NDV infection of domestic ducks involved in poultry production.

Over the past decade, previous studies have shown that genotype VII viruses, circulating predominantly in many Asian countries including China, were responsible for disease outbreaks in chicken flocks [2], [28], [29]. Phylogenetic analysis in this study revealed that the SD09 strain had highest similarity (97.78%) to the GM isolate (Accession number DQ486859), which is a classic genotype VII virus from chickens in China. The close phylogenetic proximity between SD09 and GM suggests that viral transmission may occur among chickens and domestic ducks, although further investigation is required. In addition, GD09-2 strain was found to be a genotype IX virus, which has been seldom isolated from chicken flocks in recent years. The results further indicate that genotype IX viruses still causes sporadic infections in domestic ducks in China.

In summary, we have demonstrated that there is more than one genotype of NDV circulating in the domestic ducks of China, and some strains have obvious pathogenicity to domestic ducks suggesting that ducks may play an important role in driving the evolution of NDVs. More studies are needed to further clarify the relationship and origin of NDVs in chickens and domestic ducks.

## Materials and Methods

### Viruses and animals

Two NDV isolates were recovered from diseased duck flocks in China in 2009, in which the infected ducks manifest a clinical symptom with egg drop and sporadic mortality. The strains were designated NDV/Duck/China/GD09-2/2009 (abbreviated as GD09-2) and NDV/Duck/China/SD09/2009 (abbreviated as SD09) respectively. The two viruses were purified three times using a plaque technique before being propagated in the allantoic cavities of 10-day-old specific pathogen free (SPF) embryonated chicken eggs. Virus stocks were stored at −80°C until use. To investigate the pathogenicity of two NDV isolates, SPF chickens and NDV antibody-negative Beijing ducks from Beijing Golden Star Duck Centre were used. All animal research was approved by Beijing Administration Committee of Laboratory Animals under the leadership of the Beijing Association for Science and Technology, the approve ID is SYXK (Beijing) 2007-0023.

### Assessment of the biological characteristics of two isolates

The pathogenic potential for the two isolated viruses was evaluated using standard assay methods to determine the MDT in 10-day-old chick embryos and the ICPI in 1-day-old chicks [5]. The 50% embryo infectious doses (EID_50_) of two isolates was also determined with 10-day-old chick embryos and calculated by the method of Reed and Muench.

### Primer design

Based on the available NDV nucleotide sequences (SF02, FP1/02, ZJ1, F48, JS/1/97/Ch and ZJ/1/86/Ch, with GenBank accession numbers AF473851, FJ872531, AF431744, FJ436302, FJ436305 and FJ436303, respectively), 17 pairs of specific primers were designed to amplify the complete genome of SD09 and GD09-2, excluding the 5′ and 3′ terminal segments. All primers used for this study are listed in Table 2.

### Total RNA extraction and reverse transcription-polymerase chain reaction (RT-PCR)

Viral genomic RNA was extracted from allantoic fluid using Trizol reagent (Invitrogen, San Diego, USA) according to the manufacturer's instructions. Reverse transcription was performed at 37°C for 1 h using 3 µg total RNA, 1 µL random primers (500 µg/mL random hexadeoxynucleotides) (Promega, Madison, WI, USA) and 0.5 µL M-MLV reverse transcriptase (200 U/µL) (Promega). The PCRs were performed in a thermocycler (Biometra, Germany) with 100 ng cDNA as template in a 20 µL reaction volume containing 10 pmol of each primer and 1 U Taq DNA polymerase (Promega). Reactions were performed according to the following protocol: 95°C for 5 min, followed by 35 cycles of 95°C for 45 s, 53°C or 55°C for 45 s, 72°C for 2 min, and a final elongation step of 10 min at 72°C [30]. PCR products were examined by electrophoresis on a 1.5% (w/v) agarose gel and visualized after Goldview staining.

### Cloning and sequencing of PCR products

PCR products of the expected length were purified with a Gel Extraction kit (OMEGA, USA), then cloned into the PMD18-T vector (TaKaRa, Japan) according to the manufacturer's instructions and sequenced at BGI (Beijing, China). At least three clones of each segment were sequenced to control for Taq DNA polymerase misincorporation errors.

### Phylogenetic analysis

Complete NDV genomic sequences were obtained from GenBank (Table 3), and these included current vaccine strains, typical prevailing isolates in China and the reference strains for each known NDV genotype. These NDV sequences and the complete coding sequences of the two NDV isolates were aligned and analyzed using the ClustalW multiple alignment algorithm in the MegAlign program of the DNASTAR software suite (version 3.1; DNAstar, Madison, WI, USA).

A phylogenetic tree was constructed using MEGA4.0 software (Molecular Evolutionary Genetics Analysis, version 4.0) by Neighbor-Joining method (1000 replicates for bootstrap). The evolutionary distances were computed by Pairwise Distance method using the Maximum Composite Likelihood Model [31].

### Clinicopathologic assessment in chickens

Three groups, each containing ten 1-week-old SPF White Leghorn chickens were inoculated *via* the intranasal route with 0.3 mL of one of the viruses (SD09 or GD09-2) or phosphate-buffered saline (PBS) as a non-infected control. Each bird received approximately 10^5.0^ (SD09) or 10^7.0^ (GD09-2) EID_50_ of viral inoculum based on titrations in embryonated eggs to confirm the administered dose.

All birds were monitored clinically every day for signs of disease (disheveled feathers, lethargy, fever or paralysis) and mortality. Tissues (trachea, lung, brain, spleen, small intestine, proventriculus and kidney) were collected and fixed by immersion in 10% neutral buffered formalin for approximately 72 h, then 3 µm sections were prepared for histological observation.

### Clinicopathologic assessment in ducks

Thirty 1-week-old Peking NDV antibody-negative ducks were randomly divided into three groups. The challenge procedure was carried out as described in the chicken experiments with slight modifications. All ducks were monitored daily for two weeks. Serum samples were collected from all birds before inoculation and at 14 days post infection (dpi) for NDV-specific antibody detection by a hemagglutination inhibition (HI) test in microtiter plates with 1% chicken red blood cells. Tissues (liver, spleen, small intestine and kidney) were collected for histological observation as described. To confirm the pathology in ducks, antigens of NDV in liver of GD09-2 inoculation group was further examined by immunohistochemistry (IHC) which employed an HN-protein-specific mouse monoclonal antibody. To test for virus shedding, cloacal swab samples were collected from all birds in GD09-2 group on 1, 3, 5 and 7 dpi to detect viral RNA by RT-PCR as previously described [2], [31].
